# Assessment of Oral Health Education with the Simplified Oral Hygiene Index in Military Students – A Comparative Study

**DOI:** 10.3290/j.ohpd.b1993907

**Published:** 2021-09-11

**Authors:** Ancuța Dumitrița Dan, Doina Lucia Ghergic

**Affiliations:** a PhD Student, Doctoral School – Dental Medicine, Titu Maiorescu Univesity, Bucharest, Romania. Contributed to developing the study plan, design, and implementation, designed the educational intervention, supported initial and follow-up evaluations and data collection, prepared the manuscript, approved the final draft, finalised the paper for submission.; b Professor, Doctoral School – Dental Medicine, Titu Maiorescu Univesity, Bucharest, Romania. Contributed to developing the study plan, design, and implementation, designed the group educational intervention, delivered the educational intervention to the participants, analysed the data; approved the final draft.

**Keywords:** dental hygiene counseling, oral health, patient education, Simplified Oral Health Index, young adults

## Abstract

**Purpose:**

To assess the impact of participation in a group oral health education course on oral hygiene in Romanian military students compared to a non-participant control group.

**Materials and Methods:**

A sample of 318 participants was enrolled from 805 recruited students. Baseline and 6-month post-intervention Simplified Oral Hygiene Index (OHI-S) scores were compared between an oral health education intervention (OHE) group (N = 159) and a control group (N = 159) using Student’s t-test. All participants received individualised instruction; only those in the OHE group participated in interactive group oral-health training.

**Results:**

Post-intervention OHI-S scores improved statistically significantly (p < 0.05) in both groups compared to baseline scores. The OHE group’s post-intervention OHI-S scores were statistically significantly better (p < 0.05) than the control group’s intervention scores. Women had better OHI-S scores than men at both time points.

**Conclusions:**

An interactive educational module produced favorable oral health results. It would be appropriate to provide an oral health course to military students aimed at supporting the maintenance of good oral health.

The maintenance of a good oral status in active duty personnel supports a high combat level and reduces the risk of dental emergencies during combat missions, especially in isolated and non-evacuable areas without easy access to specialised medical facilities, such as in long-term submarine missions, space missions, military exercises in remote locations, and deployments of military forces and non-governmental organisations in conflict zones. Problems related to dental emergencies that would be difficult to treat without medical evacuation can jeopardise the success of military operations due to both economic and strategic costs. The evacuation of a soldier with a dental emergency requires up to nine accompanying soldiers and either a three-car military convoy or an aeronautical medevac.^7^

Conditions that require urgent curative interventions include, most frequently, caries and secondary caries, followed by periodontal pathology, dental fractures, and endodontic pathology.^19^ These conditions can be prevented with adequate oral hygiene routines,^12^ rigorous periodic management and ongoing health education of service members beginning in military education institutions.^31^

Such education should include a good toothbrushing technique, dental and oral hygiene, and oral hygiene self-assessment skills. Education plans may include theoretical training in a group setting as well as one-on-one demonstrations. Moreover, it has been suggested that these two components of education should be followed by repeated assessments to assess the effectiveness of the education program.^8,18,32^

The military aims of obtaining and maintaining fighting ability readiness are dependent on troops’ sustaining optimal levels of general health, including oral health. Health maintenance among military personnel is supported by continuous physical and mental training, proper nutrition, and adequate rest.^4,13,16,28^ To support military preparedness and guide craniofacial protective measures, Lee et al^17^ published a review of studies of individual medical readiness among US military personnel, with a focus on dental readiness, encompassing data ranging from 1955 to 2017. They found that, on average, some 12% of troops in hostile environments will have a dental emergency or experience a cranial/oral maxillofacial injury requiring urgent surgery.^17^

NATO (North Atlantic Treaty Organization) established a protocol of oral health standards for its member states that military personnel must meet to be considered fit to participate in NATO missions, and mission participation fitness is determined based on a NATO classifcation system of oral health status.^20^ Romania is a strategic NATO member and an active participant in multinational exercises and operations. However, oral health status data for Romanian military students and active duty personnel are lacking in the literature. Moreover, there is no national Romanian health policy aimed at improving the oral health of young adults and no particular focus on oral health improvement by military decision-makers in charge of setting medical expectations. Beyond standard health examinations, which include dental check-ups, the Romanian government has no ongoing educational programs to combat oro-dental diseases in active duty personnel, including military students and combatants.

Thus, the aims of this randomised, blind interventional study were twofold: (1) to obtain accurate recent data on the oral health status of Romanian military students, as reflected by Simplified Oral Hygiene Index (OHI-S) scores, and (2) to evaluate the impact of attending an oral health course focused on healthful behaviours and habits of Romanian military students. Data were compared between an experimental group of military students who participated in the oral health education course (OHE group) and a control group who did not. Both groups received individual one-on-one oral health training. The hypothesis of this research was that the OHE group would have better OHI-S scores than those who received only individual oral health training.

## Materials and Methods

### Ethics Approval

All methods involving human participants were carried out in accordance with relevant guidelines and regulations as well as the 1964 Helsinki Declaration and its later amendments. This study was reviewed and approved by the Ethics Committee at the Faculty of Dental Medicine, Titu Maiorescu University, and by the commander of the Ferdinand I Military Technical Academy (CR 1115/20.02.2019). Written informed consent was obtained from all individual participants included in the study.

### Participants

A convenience sample of 805 students (569 males and 236 females; mean age = 20.1 years, standard deviation = 1.25) enrolled at Ferdinand I Military Technical Academy (the Academy from here on), a polytechnic university for Romanian defense systems, were recruited to participate in this study, including 327 first-year students, 259 second-year students, and 219 third-year students. The inclusion criteria were active student status in year one, two, or three at the Academy and provision of written informed consent for voluntary participation in a research study.

A power analysis was conducted in OpenEpi^23^ with the following parameters: outcome factor proportion of 0.5, a type I error rate allowance of 0.05, and a confidence level of 95%. Maximum variance was calculated as σ(p) = p(1 - p), where σ represents the standard deviation and p is probability of attaining a type II error, with a margin of error Δ(p) of ±5 p.p. Under these conditions, an N of at least 261 students was recommended to ensure a representative sample.

### Study Design

All participants were clinically evaluated with the Simplified Bacterial Plaque Index (BPI-S) and the Simplified Tartar Index (TI-S). OHI-S values were determined for each participant at the outset of the study immediately before the intervention (baseline) and 6 months after the intervention (post-intervention). Based on this baseline score, individualised training was offered to each participant to improve oral hygiene. This individualised training, which was performed during the initial clinical evaluation, involved the patient using a mirror to visually examine the areas covered by bacterial plaque. That is, plaque differential discloser was applied to enable each participant to easily see the areas where he or she needs to practice more intensive oral hygiene. At this time, the examining doctor answered patient questions, made recommendations regarding curative treatments (e.g. fillings for caries, etc.) and dental health maintenance practices, and presented the participant with auxiliary means of hygiene appropriate for each participant’s needs (e.g. flossing options, specialised toothpastes, etc.).

Half of the enrolled students participated in an oral health education module aimed at increasing oral health knowledge and improving oral self-care skills (OHE group), while the remaining half constituted the control group. The above-mentioned clinical evaluations were repeated in a post-intervention re-assessment. The participants were assigned to the experimental OHE group or control group based on block randomisation (quadruple blocks produced in Microsoft Excel, v 2013; Redmond, WA, USA) by a nurse from the medical office of the Academy. In order to prevent bias, the examining dentist and the individual collecting data were blinded to the group allocation of participants. Also the individual performing statistical analysis was blinded by labelling the groups with non-identifying terms (group A and group B).

### Evaluations

Baseline clinical evaluations were conducted at the dentist’s office of the Academy in March and April of 2019. Post-intervention clinical re-evaluations took place in January and February of 2020, using the same methodology as in the baseline evaluation. Prior to each examination, subjects were asked to remove any removable orthodontic braces or protheses. The examination time was 5–15 min per patient (median time, 8 min). During each examination, the following supplies were used: light, air/water spray, cotton rolls, cotton balls, dental plaque disclosing gel (GC Tri Plaque ID Gel; Tokyo, Japan), and a consultation kit. The plaque disclosing gel contains sucrose and pigments (red and blue) that penetrate and affix to plaque biofilm, providing a color-coded demonstration that can be used to educate patients about their plaque status. Briefly, new/sparse areas of plaque appear pink/red (unable to hold blue pigment), established plaque areas (>48 h old) appear blue/purple (higher density traps both pigments), and highly pathogenic plaque areas appear light blue due to acidogenic bacteria (red pigment is metabolised, leaving only blue pigment). To facilitate thorough examination, the teeth were dried for 5 s with air spray and cotton balls, and the examined teeth were illuminated by a dental-unit light source. Dental plaque disclosing gel was applied with a cotton ball to the surfaces to be examined, after which the teeth were washed with a gentle jet of water. The surfaces were evaluated in terms of the presence of bacterial plaque (quantitative and qualitative) and tartar. After the baseline examination, we performed personalised training with each subject, aimed at improving oral hygiene practices. BPI-S and (subsequently) TI-S scores were recorded for vestibular surfaces (1.6, 1.1, 2.6, 3.1) and lingual surfaces (3.6, 4.6). Scores assigned to each examined surface varied depending on the degree of spread of plaque or tartar; the scoring system used is provided in Table 1. BPI-S and TI-S values were obtained as the arithmetic means of the scores recorded for each evaluated area (at least two surfaces). BPI-S and TI-S values (range of both: 0–6) were classified as excellent (0), good (0.1–1.2), mild (1.3–3.0), or poor (3.1–6.0). The BPI-S and TI-S values were summed to obtain overall OHI-S scores (range: 0–12) for each participant, which were categorised as excellent (0), good (0.1–2.4), mild (2.6–6), or poor (6.1–12.0). During the initial clinical evaluation, each patient was shown their plaque visualisation results with a mirror, and then given instructions on how to improve their hygiene by correcting their brushing technique and using sanitation aids, which were provided.

**Table 1:** Plaque/tartar index scoring system

Score	Criteria
0	Absence of bacterial plaque/tartar
1	Supragingival bacterial plaque/tartar in 1/3 of the tooth cervical area
2	Supragingival bacterial plaque/tartar in the middle 1/3 of the tooth
3	Supragingival bacterial plaque/tartar reaches ≤ 1/3 occlusal/incisal crown

### Health Education Intervention

The oral health educational module administered to the OHE group had a well-planned framework focused on four dimensions: general information about dentition; food hygiene; oral and dental hygiene tools; and the patient-dentist relationship in order not to miss relevant information.^25^ During interactive training, in addition to the topics set to be presented in each chapter, specific information was developed in response to the participants’ interests and the most frequent pathologies detected during the initial clinical evaluation. The training module included a formal didactic session, role playing, and introductions to online supplemental materials. It concluded with a final question and answer period. The intervention module lasted 120 min. The control group did not receive this group training.

### Data Analysis

Data were compared between the OHE group and the control group using Student’s t-tests (independent sample t-tests and paired samples t-tests) in Microsoft Excel, v 2013. Stastistical significance was set at p < 0.05 in all cases.

## Results

### Participant Characteristics

Of 805 subjects recruited to participate, only 318 (39.5%) gave informed consent to participate in the study, including 133 first-year students, 88 second-year students, and 97 third-year students. The final study cohort of 318 participants exceeded the threshold for being statistically representative (OpenEpi suggested N ≥ 261). It included 231 men (72.6%) and 87 women (27.4%). The mean age of the enrolled participants was 20.2 years, ranging from 18 (N = 8, 2.2%) to 24 (N = 1, 0.3%). The highest percentage of subjects was 21 years old (N = 123, 33.9%). Demographically, 106 participants (33.3%) came from rural areas and 212 (66.7%) came from urban areas, with 108 participants (34.0%) having graduated from military high schools and 210 subjects (66.0%) having graduated from civilian high schools.

All patient/personal identifiers were removed so the participants described would not be identifiable and could not be identified based on information in this report.

### Clinical Observations

At the baseline clinical examination before the intervention, the overall mean BPI-S score obtained was 1.48 (range, 0.5–3.0), the overall mean TI-S score obtained was 0.72 (range, 0.0–3.0), and the overall mean OHI-S score obtained was 2.2 (range, 0.6–6.0). The initial clinical evaluation results are summarised in Table 2.

**Table 2:** Oral hygiene evaluation scores at baseline for oral health education intervention (OHE) and control groups, reported as mean, standard deviation (SD), and standard error (SE)

		Simplified Bacterial Plaque Index I			Simplified Tartar Index I			Simplified Oral Hygiene Index I		
Variable	N	Mean	SD	SE	Mean	SD	SE	Mean	SD	SE
Total	318	1.48	0.5	0.02	0.72	0.36	0.02	2.2	0.75	0.04
Gender										
Male	231	1.53	0.51	0.03	0.77	0.37	0.02	2.3	077	0.05
Female	87	1.34	0.45	0.04	0.58	0.26	0.02	1.93	0.63	0.06
p-value		0.001			5.61278E-07			1.29322E-05		
Origin										
Urban	212	1.49	0.49	0.05	0.71	0.36	0.02	2.2	0.74	0.05
Rural	106	1.46	0.52	0.05	0.74	0.35	0.03	2.2	0.76	0.07
p-value		0.31			0.3			0.48		
High school										
Military	108	1.47	0.49	0.04	0.73	0.33	0.03	2.21	0.73	0.07
Civilian	210	1.48	0.5	0.03	0.71	0.37	0.02	2.19	0.76	0.05
p value		0.43			0.3			0.43		
Group										
OHE	159	1.52	0.53	0.04	0.72	0.4	0.03	2.24	0.83	0.06
Control	159	1.43	0.46	0.03	0.72	0.4	0.03	2.15	0.65	0.05
p-value		0.06			0.5			0.13		

At the 6-month post-intervention follow-up evaluation, the overall mean BPI-S, TI-S, and OHI-S values obtained were 1.38 (range, 0.3–3.0), 0.70 (range, 0.0–3.0), and 2.1 (range, 0.5– 6.0), respectively. The follow-up clinical evaluation results are summarised in Table 3. Table 4 compares the OHI-S results across baseline and follow-up time points for the OHE and control groups. At the post-intervention evaluation, we observed a statistically significantly better OHI-S for the OHE group than for the control group (p = 0.03), indicating an improvement in oral hygiene especially among participants who had benefited from the interactive group health-education module. Between the two evaluation time points, the OHE group showed a statistically significant improvement in mean BPI-S score (p = 0.01), but not in mean TI-S score (p = 0.48).

**Table 3:** Oral hygiene evaluation scores at 6-month follow-up in oral health education intervention (OHE) and control groups, reported as mean, standard deviation (SD), and standard error (SE)

		Simplified Bacterial Plaque Index II			Simplified Tartar Index II			Simplified Oral Hygiene Index II		
Variable	N	Mean	SD	SE	Mean	SD	SE	Mean	SD	SE
Total	318	1.38	0.5	0.02	0.7	0.38	0.02	2.08	0.75	0.04
Gender										
Male	231	1.44	0.5	0.03	0.75	0.39	0.02	2.19	0.76	0.05
Female	87	1.23	0.46	0.05	0.57	0.3	0.03	1.81	0.67	0.07
p-value		0.0003			1.78874E-05			1.2972E-05		
Origin										
Urban	212	1.4	0.49	0.03	0.71	0.39	0.02	2.1	0.74	0.05
Rural	106	1.35	0.52	0.05	0.68	0.36	0.03	2.04	0.77	0.07
p-value		0.21			0.27			0.23		
High school										
Military	108	1.39	0.54	0.05	0.69	0.36	0.03	2.09	0.79	0.07
Civilian	210	1.38	0.5	0.03	0.7	0.39	0.02	2.08	0.73	0.05
p-value		0.44			0.4			0.46		
Group										
OHE	159	1.45	0.52	0.04	0.72	0.41	0.03	2.17	0.8	0.06
Control	159	1.32	0.47	0.03	0.68	0.34	0.02	1.99	0.69	0.05
p-value		0.01			0.12			0.03		

**Table 4:** Distribution of cases with lower (better), equal (unchanged), or higher (worse) Simplified Oral Hygiene Index (OHI) scores 6 months after the intervention compared to pre-intervention baseline values

		Final OHI < baseline OHI		Final OHI = baseline OHI		Final OHI > baseline OHI	
Variable	N	N	%	N	%	N	%
Total	318	191	60%	49	15.4%	78	24.6%
Gender							
Male	231	137	59.3%	38	16.45%	56	24.25%
Female	87	54	62.08%	11	12.64%	22	25.28%
Origin							
Urban	212	129	60.85%	29	13.68%	54	25.47%
Rural	106	62	58.5%	20	18.87%	24	22.63%
Highschool							
Military	108	60	55.55%	18	16.68%	30	27.77%
Civilian	210	131	62.4%	31	14.76%	48	22.84%
Group							
OHE	159	108	67.92%	20	18.51%	31	13.57%
Control	159	83	52.2%	29	18.24%	47	29.56%

Overall, across both groups, we observed a statistically significant improvement in OHI-S (p = 0.05). Women had better OHI-S scores than men at both time points (both p < 0.01). Overall, OHI-S did not differ statistically significantly in relation to rural/urban origin (baseline p = 0.97; post-intervention p = 0.47) or high school type (baseline p = 0.86; post-intervention; p = 0.93).

## Discussion

In the present study, we found that the OHE experimental group and control group had statistically similar BPI-S, TI-S, and OHI-S scores at baseline. Overall, at the 6-month post-intervention evaluation, OHI-S scores had improved compared to baseline values, evidence of an improvement in oral hygiene for all participants, who all received one-on-one training. These results agree with Notoatmodjo’s^21^ assertion that knowledge about health transmitted by way of health education can affect behaviour and thus long-term outcomes, as well as Widyawati’s^29^ opinion that dental and oral health education can affect attitudes related to the maintenance of oral hygiene.

At the post-intervention timepoint, the OHE group had statistically significantly better OHI-S scores than the control group, suggesting that the combined training that they received, including one-on-one instruction and an interactive group class, was more effective for improving oral hygiene than the one-on-one instruction alone. This finding is consistent with Dale’s Cone of Learning model, which suggests that information recall depends on the educational methods used.^10^ Moreover, these results support Wilson’s^30^ conclusions that both individual and group education can improve patient outcomes and that, in some cases, group education can be more effective. Although any mode of education can improve recipients’ knowledge in the short term, some educational methods may be more effective than others in the long term. The effectiveness of our interactive group education intervention in this study is consistent with results from Poland.^1^

Our findings of better (lower) index scores in women than men suggest that women, on average, may have better oral hygiene. Indeed, recruited women showed greater interest in learning about oral health and they exhibited a higher degree of interest during clinical evaluation and individual training as well as in group training (among those participants who were in the OHE group). This finding is in agreement with an earlier study conducted in Indonesia that showed female students paid more attention to dental and oral health than did male students.^27^ Similar results have also been reported from studies conducted in Japan^15^ and Uganda.^22^

Although we did not observe statistically significant differences between students from urban vs rural areas, we did observe a notable improvement in the scores of students from rural areas from the baseline to the final clinical examination, indicating that they were receptive to the educational information provided. Although health literacy and health outcomes in rural populations tend to be lower than in urban populations,^3,11,20^ rurality is typically not a specific determinant of health literacy. Education, age, gender, socioeconomic status, and race/ethnicity have been previously shown to have strong associations with health literacy and implicit health outcomes in both rural and urban areas.^2,5,6,9,14,24^ We observed statistically similar improvements in students who attended military high schools and those who attended civilian high schools.

A main limitation of this study is the low recruit-to-participant conversion rate (39.5%). The reasons given for declining to participate included busy schedules, fear of the dentist (despite understanding that no therapeutic intervention would be performed), a lack of interest in participating in scientific research, and a lack of interest in learning more about the status of one’s own oral health. Given that out of 805 students targeted, only 318 chose to participate in this study, the study’s findings do not have a high degree of generalisability. Study results may also be underestimations. Future studies are needed to confirm the findings.

## Conclusions

The intervention evaluated here, which consisted of an interactive group instruction module in addition to individual training, was confirmed to improve oral hygiene relative to individual training in the dentist’s office alone. We thus conclude that it would be appropriate to introduce an oral health education course into the education of military academy students to support the establishment of proactive healthy behaviours with favourable long-term results in terms of oral health and quality of life. This recommendation is in keeping with the notion that it is important to include health education that supports the health status of students.^26^

### Strengths and Limitations of the Study

The implementation of methods to improve oral hygiene is an important issue that affects dental health and ultimately either strengthens or undermines force readiness.This study provided a better understanding of how oral hygiene of military students is influenced by different oral health training methods. These documented experiences can be useful for establishing oral health policy and oral health education programs. A limitation of the study is that it was conducted at a single Romanian military academy (one of the five existing) which can affect its generalisability.

### Practice Implications

This study highlights the benefits of oral health and dental hygeine training, particularly for young adult military students. Additionally, the results confirm the usefulness of bacterial plaque detectors in in-office individual training sessions.
